# Rab5b-Associated Arf1 GTPase Regulates Export of N-Myristoylated Adenylate Kinase 2 From the Endoplasmic Reticulum in *Plasmodium falciparum*

**DOI:** 10.3389/fcimb.2020.610200

**Published:** 2021-02-02

**Authors:** Izumi Taku, Tomohiro Hirai, Takashi Makiuchi, Naoaki Shinzawa, Shiroh Iwanaga, Takeshi Annoura, Kisaburo Nagamune, Tomoyoshi Nozaki, Yumiko Saito-Nakano

**Affiliations:** ^1^ Department of Parasitology, National Institute of Infectious Diseases, Tokyo, Japan; ^2^ Graduate School of Life and Environmental Sciences, University of Tsukuba, Ibaraki, Japan; ^3^ Department of Parasitology, Tokai University School of Medicine, Isehara, Japan; ^4^ Department of Environmental Parasitology, Graduate School of Medical and Dental Sciences, Tokyo Medical and Dental University, Tokyo, Japan; ^5^ Faculty of Life and Environmental Sciences, University of Tsukuba, Ibaraki, Japan; ^6^ Graduate School of Medicine, The University of Tokyo, Tokyo, Japan

**Keywords:** *Plasmodium falciparum*, Rab5b, Arf1, Rab1b, AK2, Rifin, ERD2

## Abstract

*Plasmodium falciparum* extensively remodels human erythrocytes by exporting hundreds of parasite proteins. This remodeling is closely linked to the *Plasmodium* virulence-related functions and immune evasion. The N-terminal export signal named PEXEL (*Plasmodium* export element) was identified to be important for the export of proteins beyond the PVM, however, the issue of how these PEXEL-positive proteins are transported and regulated by Rab GTPases from the endoplasmic reticulum (ER) to the cell surface has remained poorly understood. Previously, we identified new aspects of the trafficking of N-myristoylated adenylate kinase 2 (PfAK2), which lacks the PEXEL motif and is regulated by the PfRab5b GTPase. Overexpression of PfRab5b suppressed the transport of PfAK2 to the parasitophorous vacuole membrane and PfAK2 was accumulated in the punctate compartment within the parasite. Here, we report the identification of PfRab5b associated proteins and dissect the pathway regulated by PfRab5b. We isolated two membrane trafficking GTPases PfArf1 and PfRab1b by coimmunoprecipitation with PfRab5b and *via* mass analysis. PfArf1 and PfRab1b are both colocalized with PfRab5b adjacent to the ER in the early erythrocytic stage. A super-resolution microgram of the indirect immunofluorescence assay using PfArf1 or PfRab1b- expressing parasites revealed that PfArf1 and PfRab1b are localized to different ER subdomains. We used a genetic approach to expresses an active or inactive mutant of PfArf1 that specifically inhibited the trafficking of PfAK2 to the parasitophorous vacuole membrane. While expression of PfRab1b mutants did not affect in the PfAK2 transport. In contrast, the export of the PEXEL-positive protein Rifin was decreased by the expression of the inactive mutant of PfRab1b or PfArf1. These data indicate that the transport of PfAK2 and Rifin were recognized at the different ER subdomain by the two independent GTPases: PfAK2 is sorted by PfArf1 into the pathway for the PV, and the export of Rifin might be sequentially regulated by PfArf1 and PfRab1b.

## Introduction

Secretory proteins are synthesized in the endoplasmic reticulum (ER) and are delivered to their destinations *via* the Golgi apparatus. ER-to-Golgi trafficking is highly conserved among eukaryotes, and two types of Ras super families of GTPases, the Sar/Arf and Rab families, mediate protein sorting and transport as molecular switches between these organelles (Suda et al., 2018; Kurokawa and Nakano, 2019). A guanine nucleotide exchange factor (GEF) acts on GDP-bound form of GTPase to convert it to a GTP-bound active state, and a GTPase accelerating protein (GAP) binds to the GTPase to catalyze hydrolysis of the bound GTP to GDP and thereby convert the GTPase back to its inactive state (Hutagalung and Novick, 2011). The GTPase cycle between GDP-and GTP-bound forms causes conformational changes and specific effector molecules are recruited to the GTP-bound active form to complete various membrane trafficking events (Stenmark, 2009; Donaldson and Jackson, 2011). Many regulatory functions performed by the proteins in the Sar/Arf and Rab families were identified by their interaction with diverse effector proteins that select cargo, promote vesicle movement, and verify the correct site for fusion (Hutagalung and Novick, 2011).

The Sar/Arf and Rab families of GTPases regulate the bidirectional transport between ER and the Golgi in mammalian cells and land plants (Brandizzi and Barlowe, 2013). Newly synthesized secretory proteins translocate into the ER lumen using the N-terminal signal peptide (Nickel and Rabouille, 2009). The ER luminal chaperone BiP assists in folding newly synthesized proteins to maintain protein homeostasis (Craig et al., 1993). Activation of the ER-localized GTPase Sar1 and its GTP hydrolysis triggers the recruitment of the COPII coat component and causes the enrichment of cargo proteins into the ER exit sites (ERES) where COPII-coated vesicles form (Kung et al., 2012; Yorimitsu and Sato, 2012). The COPII machinery performs two critical functions: first, Sar1 and the inner layer Sec23–Sec24 COPII subunits bind to and select specific cargo for packaging; second, polymerization of the outer layer of Sec13–Sec31 COPII subunits occurs into a cage structure to drive vesicle formation (Bi et al., 2007). After uncoating of the COPII coat, diffusive vesicles are anchored to *cis*-Golgi membranes by the extended coiled-coil domain that tethers factor protein p115, the yeast homolog of Uso1, as well as the multi-subunit TRAPPI (transport protein particle I) complex that activates Rab1 GTPase (Cai et al., 2007). The fusion of vesicles depends on the assembly of integral membrane SNARE complexes between the donor and acceptor membranes (Allan et al., 2000). In contrast, a heptameric complex COPI-coated vesicle facilitates the retrieval of escaped ER luminal proteins that contain KDEL signals, which are recognized by the resident KDEL receptor ERD2 in the *cis*-Golgi, and by SNARE proteins (Lewis et al., 1990; Semenza et al., 1990). Once Arf1 is activated by the GEF which contains a conserved Sec7 domain, the membrane-localized Arf1 recruits the COPI coat to the *cis*-Golgi membranes (Zhao et al., 1999). The COPI subunits recognize the sorting motifs of transmembrane cargo proteins and are incorporated into nascent COPI vesicles and subsequently retrieve cargo proteins to the ER (Eugster et al., 2000). Most Rab and Arf GTPases carry lipid modifications that are necessary for membrane recruitment. In the case of Rab, a carboxy-terminal geranylgeranylation (20-carbon unsaturated fatty acid) group at the C-terminal cysteine residue is responsible for the membrane attachment of the Rab-GTP form (Leung et al., 2006). In contrast, the N-terminal glycine residue of Arf is myristoylated (14-carbon saturated fatty acids) (Pasqualato et al., 2002).

*Plasmodium falciparum* is the causative agent of malaria, and it extensively remodels the human erythrocytes in which it resides (de Koning-Ward et al., 2016). The remodeling process is conducted by hundreds of proteins exported from the parasites into the host cell compartment, and these enable virulence-related functions including cytoadherence to the vascular endothelium, immune evasion, and nutrient uptake (Miller et al., 2013). Many of the exported proteins contain a five amino acid motif termed the *Plasmodium* export element (PEXEL) which is found ~20 amino acids downstream of the signal peptide (Hiller et al., 2004; Marti et al., 2004). The PEXEL motif is removed in the ER lumen of the parasite by the ER resident aspartic protease plasmepsin V before exit of the ER (Boddey et al., 2010; Russo et al., 2010). All repertoires of COPII and COPI components are conserved in the *Plasmodium* spp. (Kibria et al., 2019), suggesting that the early secretory system of *Plasmodium* may resemble that in higher eukaryotes. Several studies have reported that the fungal metabolite brefeldin A (BFA), which inhibits the Golgi-localized GEF Arf1, disrupts the accurate export of PEXEL-containing proteins (Akompong et al., 2002; Chang et al., 2008), indicating that these proteins are trafficked through the classical ER-Golgi pathway. Subsequently, the exported proteins are transported across the parasite plasma membrane and the parasitophorous vacuole membrane (PVM) *via* the multimeric *Plasmodium* translocon of exported proteins (PTEX) into the erythrocyte cytosol (de Koning-Ward et al., 2009). In these *Plasmodium* trafficking systems, the roles of small GTPases are poorly understood such that the subcellular localization and function of only one Sar1 and six Rab GTPases has been being characterized functionally; i.e., PfSar1, PfRab1a, PfRab5a, PfRab5b, PfRab6, PfRab7, and PfRab11A (Adisa et al., 2001; Struck et al., 2005; Elliott et al., 2008; Agop-Nersesian et al., 2009; Rached et al., 2012; Krai et al., 2014; Ebine et al., 2016; Morse et al., 2016). However, effectors and binding proteins have only been identified for PfRab11A (Agop-Nersesian et al., 2009). Further, PfRab11A is associated with the myosin-tail-interacting-protein and is crucial for the parasite invasion of host cells (Agop-Nersesian et al., 2009).

Several exported proteins lacking an N-terminal PEXEL motif and are called PEXEL-negative proteins (PNEPs). These are also translocate through PTEX for export into the erythrocyte cytosol (Spielmann and Gilberger, 2010; Elsworth et al., 2014). For example, the skeleton binding protein 1 (SBP1), which is a PEXEL-negative transmembrane protein, is supposed to export *via* the classical secretory pathway, because the truncated SBP1 internalized in the perinuclear staining corresponding to the ER (Saridaki et al., 2009). However, recent report that SBP1 was included within electron-dense materials in the parasite cytoplasm (Iriko et al., 2020), strongly suggests that the presence of alternate export pathways except for the classical ER-to-Golgi transport pathway in the parasite trafficking system. *Plasmodium falciparum* adenylate kinase 2 (PfAK2), which lacks a signal peptide and the PEXEL motif, but is modified *via* myristoylation (14-carbon) and palmitoylation (16-carbon saturated fatty acid) at the N-terminal glycine and cysteine residues respectively, is transported to the parasite PVM (Rahlfs et al., 2009; Thavayogarajah et al., 2015; Ebine et al., 2016). Previously, we have reported that trafficking of PfAK2 to the PVM was repressed and the PfAK2 accumulated in the punctate structure within the parasite cytoplasm due to the overexpression of PfRab5b, indicating that PfRab5b is involved in the transport of PfAK2 to the PVM (Ebine et al., 2016). PfRab5b possesses a structurally unique motif that is similar to PfAK2, which lacks a C-terminal geranylgeranylation modification, while retaining the N-terminal myristoylation and palmitoylation motifs (Ezougou et al., 2014). In rodent malaria *P. berghei*, gene-deletion of PbRab5b was unsuccessful, indicating *Plasmodium* Rab5b is essential for the blood stage growth (Ezougou et al., 2014). Subcellular localization of PfRab5b is proximal to the region of the BiP-positive ER, and segregated from COPII subunit Sec13-positive ERES (Ebine et al., 2016). Interestingly, the inhibition of transport was observed in PfAK2, however the PEXEL-positive erythrocyte vesicle protein 1 (PfEVP1) and SBP1 were correctly exported upon the overexpression of PfRab5b (Ebine et al., 2016). These findings suggested that PfRab5b may regulate the transport of PfAK2 by the COPII independent non-classical pathway (Ebine et al., 2016).

Currently, none of GTPase, which regulates the trafficking of PNEPs including PfAK2, is elucidated, A bioinformatic technique previously identified casein kinase1 (CK1) as a PfRab5b binding protein (Rached et al., 2012); however, it remains unclear how PfRab5b is involved in the selective transport of PfAK2. To understand the mechanisms underlying the PfRab5b-dependent specific trafficking pathway, we isolated PfRab5b associated proteins using coimmunoprecipitation and mass analysis approaches. Among the candidate proteins, we found that the PfArf1 and PfRab1b GTPases were colocalized with PfRab5b in the compartment close to the ER. Indirect immunofluorescence assay for PfArf1- or PfRab1b- expressing parasites using super-resolution microscopy revealed that PfArf1 and PfRab1b were localized to different ER subdomains. Further, using a genetic approach to express an active or inactive mutant indicated that PfArf1, but not PfRab1b, was involved in the transport of PfAK2 to the PVM. Unexpectedly, PfRab1b participates in the trafficking of the PEXEL-positive export protein Rifin. Our data suggest that the trafficking pathways of PfAK2 and Rifin are separated in the PfArf1-positive ER subdomain, and this is the first report for the identification of GTPase which regulates transport of PfAK2 in *Plasmodium*.

## Materials and Methods

### Ethics Statement

Human RBCs and plasma were obtained as donations from anonymized individuals at the Japanese Red Cross Society (no. 28J0023).

### Strain Culture and Transfection

*Plasmodium falciparum* line MS822 (Nakazawa et al., 2011) was cultured as described previously (Alexandre et al., 2011), and transfection was performed as described (Deitsch et al., 2001; Alexandre et al., 2011). Transfected parasites were selected with 5 nM of WR99210-HCl (a kind gift from D Jacobus) or 2.5 µg/ml of blasticidin S-HCl (BSD, Funakoshi). Transfectants were selected 2 weeks after the addition of drugs.

### Plasmid Construction

The vector pCHD43(II) (Sakura et al., 2013; Ebine et al., 2016) was used to express PfRab5b-yellow fluorescent protein (YFP)-FLAG (Watanabe et al., 2020), PfRab5b-YFP, and PfRab5b-YFP-destabilization domain (DD) (Armstrong and Goldberg, 2007) from the constitutive *P. falciparum* chloroquine resistance transporter promoter. To construct PfRab5b-YFP/pCHD43(II), the DD domain was removed from the PfRab5b-YFP-DD/pCHD43(II) construct using the PrimeSTAR Mutagenesis Basal Kit (TaKaRa Bio). A tandem repeat of the FLAG tag was inserted after the YFP coding region of PfRab5b-YFP/pCHD43(II) using PCR amplification with overlapping oligonucleotides to construct PfRab5b-YFP-FLAG/pCHD43(II) (**Supplementary Figure 1A**). The artificial centromere plasmid PfCenV-ef1-double, whose expression was regulated under the *Plasmodium berghei* elongation factor 1 (Pbef1α) and maintained by the *P. falciparum* centromere of chromosome 5, was used to stably express PfRab5b and PfArf1 or PfRab1b (Iwanaga et al., 2012). Fusion construct of PfRab5b^Q94L^-YFP-DD was inserted into the NcoI site, and PfArf1-red fluorescent protein (RFP) or RFP-PfRab1b encoding genes were ligated into the NdeI site of PfCenV-ef1-double, respectively (**Supplementary Figure 1B**). For expression of AK2-RFP or Rifin-RFP, fragments were inserted into the NdeI site of PfCenV-ef1-double. The AK2-RFP fragment was amplified from PfAK2-RFP/pCHD43(II)-BSD. A fragment of Rifin (PlasmoDB accession number PFA0745w) was amplified from gDNA from the *P. falciparum* 3D7 line (Walliker et al., 1987). The fusion construct consisting of PEXEL signal (1–51 aa) of the Rifin-RFP-transmembrane domain (102–336 aa) has been described previously (Marti et al., 2004). PfArf1 and PfRab1b fragments were amplified using cDNA from the *P. falciparum* 3D7 line and the episomal plasmids expressing PfArf1-YFP-DD or DD-YFP-PfRab1b were inserted into pCHD43(II)-BSD (Ebine et al., 2016). To construct constitutively active or inactive PfArf1 (Q71L and T31N) and PfRab1b (Q67L and S22N) mutants, the PrimeSTAR Mutagenesis Basal Kit was used to introduce point mutations (**Supplementary Figure 1C**). The former substitution at specific sites in the guanine nucleotide consensus domains of human Ras^Q61L^ impaired GTP hydrolysis activity (Haubruck and McCormick, 1991), and the latter substitution of human Ras^S17A^ alter the guanine nucleotide binding affinity for GTP to GDP (Feig and Cooper, 1988), respectively. The oligonucleotides used are listed in **Supplementary Table 1**.

### Coimmunoprecipitation Assay and Mass Spectrometry Analysis

Transgenic parasites carrying the PfRab5b-YFP or PfRab5b-YFP-FLAG with pCHD43(II) episomal plasmids were cultured in 250 ml medium (5% hematocrit, 7% parasitemia), and infected red blood cells (iRBCs) were collected by centrifugation at 560 × g for 5 min and permeabilized using 0.075% saponin in phosphate buffered saline (PBS) for 30 min on ice. Samples were crosslinked using 0.5 mM 3,3’-dithiodipropionic acid di (N-hydroxysuccinimide ester) (DSP) (Sigma-Aldrich, St. Louis) for 30 min at room temperature, and were subsequently quenched by the addition of 50 mM Tris-HCl (pH 7.5) according to the manufacturer’s protocol. Next, the samples were solubilized with 250 µl of 1.0% Triton X-100 in PBS and kept on ice for 20 min. The insoluble fraction was removed by centrifugation at 9,100 × g for 5 min, and the supernatant fractions were incubated with Protein G Sepharose (GE Healthcare) at 4°C for 60 min to reduce non-specific binding during coimmunoprecipitation. The PfRab5b protein complex was immunoprecipitated using anti-FLAG antibody conjugated agarose (EZview Red Anti-FLAG M2 Affinity Gel, Sigma-Aldrich) at 4°C for 3.5 h, and the immunoprecipitated was washed thrice with 1.0% Triton X-100 in PBS. The PfRab5b protein complexes were eluted with 50 µl of 0.2 mg/ml FLAG peptide (Sigma-Aldrich) in PBS containing 50 mM DTT and 10 mM EDTA at 4°C for 16 h. The eluted proteins were separated *via* 12% SDS-PAGE and visualized using silver staining. The in-gel trypsin digestion of proteins, liquid chromatography, and time-of-flight tandem mass spectrometry (LC-ToF MS/MS) (Orbitrap, Thermo Fisher Scientific, Waltham, MA, USA) were performed as described previously (Makiuchi et al., 2013). The quantitative value, normalized with unweighted spectrum counts, was used to estimate relative quantities of proteins in the samples. Specific binding proteins were selected by the following criteria: 1) peptide fold enrichment that showed >3.5 higher in PfRab5b-YFP-FLAG samples compared with those in the PfRab5b-YFP control were selected. 2) Protein with >3.5 higher in PfRab5b-YFP-FLAG samples with no value in the PfRab5b-YFP control were selected. 3) Proteins those involved in cytosolic proteins such as proteasome and ribosomal proteins were removed from the list.

### Indirect Immunofluorescence Assay

Transgenic parasites carrying the PfRab5b, PfArf1, or PfRab1b mutant proteins fused with a DD system with artificial centromere plasmid PfCenV-ef1a-double were stabilized with 0.5 µM Shld1 for 24 h (Clontech) (Armstrong and Goldberg, 2007). Next, cultures were synchronized by the 5% sorbitol for 10 min at room temperature. After washing, ring-rich parasites were cultured until indicated erythrocytic stages under the 0.5 µM Shld1. Infected erythrocytes were collected and fixed with 4% paraformaldehyde (Thermo) in PBS at 4°C for more than 12 h. Fixed samples were permeabilized with 0.5% Triton X-100 in PBS for 30 min and blocked with 3% skim milk prepared with PBS for 10 min. Primary antibodies were used at the following dilutions: anti-BiP (1:100, kindly gift from Prof. Kita) and anti-ERD2 (1:100) (Elmendorf and Haldar, 1993). Alexa 488-conjugated anti-mouse IgG (1:10,000, Molecular Probes) was used as the secondary antibody. The number of parasites that showed colocalization of the RFP and PfERD2 or BiP signals was counted in 20–30 trophozoites from three independent experiments. A test for statistical significance was performed using the Student *t*-test.

### Reciprocal Coimmunoprecipitation and Immunoblot Analysis

Transgenic parasites carrying the PfArf1-RFP, or PfArf1-RFP and PfRab5b^Q94L^-YFP-DD with artificial centromere plasmids PfCenV-ef1a-double were cultured in 250 ml medium (5% hematocrit, 5.4% parasitemia), and collected iRBCs were permeabilized with saponin-PBS. The iRBCs were crosslinked with 2 mM DSP in 500 µl PBS for 30 min at room temperature with rotation. Samples were quenched with 50 mM Tris-HCl (pH7.5) for 15 min at room temperature with rotation, and then washed two times with 50 mM Tris-HCl (pH7.5). The sample pellets were resuspended in 500 μl lysis buffer (50 mM Tris-HCl, 150 mM NaCl, Complete protease inhibitor (Roche), 1% TritonX-100, (pH7.5) and homogenized by 30-times pipetting subsequently incubated on ice for 20 min. After unbroken cells were removed by centrifugation at 2,000 × g for 5 min, the supernatants were transferred to new tubes and incubated with 20 μl Protein G Sepharose (Sigma-Aldrich) for 30 min at room temperature to reduce non-specific binding during coimmunoprecipitation. After centrifugation at 800 × g for 5 min, the supernatant was reacted with 5 μg rabbit anti-RFP polyclonal antibody (GeneTex, GTX127897) at 4°C for over night and precipitated with 30 μl Protein G Sepharose for 1 h at room temperature. The Protein G Sepharose was washed with 1 ml lysis buffer for three times, and protein complexes were eluted with 40 µl of SDS sample buffer (250 mM Tris-HCl, 8% SDS, 8% 2-mercaptoethanol, 40% glycerol, 0.00 4% bromophenol blue, pH 6.8) at 95°C for 5 min. Bound proteins were eluted and loaded on 12% SDS-PAGE gels, followed by immunoblotting using mouse anti-GFP monoclonal antibody (1:100, Merck, 11814460001, clone 7.1 and 13.1) and mouse anti-RFP monoclonal antibody (1:200, Cell Biolabs AKR-021, clone RF5R), mouse anti-Hsp90 monoclonal antibody (1:500, Sigma, clone AC-16), and anti-mouse IgG conjugated HRP-linked antibody (1:10000, Cell Singling, 7076S).

### Microscopy Techniques

Images were acquired using an LSM700 or LSM780 confocal laser-scanning microscope (Zeiss, Germany). For distance measurements between PfBiP and PfArf1 or PfRab1b, super-resolution imaging was performed using the Zeiss LSM880 with Airyscan confocal laser-scanning microscope, that is equipped with an oil-immersion 100× objective lens (alpha Plan-Apochromat 100×/1.46 oil DIC M27 Elyra) (Zeiss, Germany). The background fluorescence from the non-transfected parasites was set as baseline. All images were acquired in the same laser voltage and gain (exposure time). Raw data were processed using the Zeiss Zen2 software to measure fluorescent intensities. The images were analyzed using the Zeiss Zen2 software or Fiji-ImageJ software (Schindelin et al., 2012). Scoring of images was judged by the blinded two experienced microscopists.

## Results

### Coimmunoprecipitation of PfRab5b-YFP-FLAG to Isolate PfRab5b Associated Candidate Proteins

Previous studies have shown that PfRab5b is localized adjacent to the ER and is involved in the trafficking of the N-myristoylated protein AK2 to the PVM (Ebine et al., 2016). In general, transport of newly synthesized transmembrane or luminal proteins from the ER is regulated by the Sar1 family of GTPases, but not by the Rab family (Nakano and Muramatsu, 1989). Proteins are packed into COPII-coated vesicles at the ERES and trafficked to the *cis*-Golgi cisternae under the control of the Rab1 and Arf1 GTPases (Balch et al., 1992; Martinez et al., 2016). To investigate the mechanism controlled by PfRab5b from the ER, we attempted to identify PfRab5b interacting proteins by coimmunoprecipitation. Lysates prepared from PfRab5b expressing parasites, which were C-terminally fused with the YFP and FLAG-tags (PfRab5b-YFP-FLAG) or a negative control PfRab5b-YFP (**Supplementary Figure 1A**), were immunoprecipitated with an anti-FLAG antibody and eluted using the FLAG peptide. The eluted samples were analyzed *via* LC-ToF MS/MS, and 677 peptides were identified from the PfRab5b-YFP-FLAG expressing lysate (**Supplementary Table 2**). Six candidate proteins showing a 3.5–fold change in the expression of PfRab5b-YFP-FLAG relative to PfRab5b-YFP were obtained. These included the ADP-ribosylation factor (PfArf GTPase) (PF3D7_1020900), erythrocyte binding antigen-181 (EBA181) (PF3D7_0102500), protein transport protein SEC7 (PfSec7) (PF3D7_1442900), early transcribed membrane protein 10.2 (ETRAMP10.2) (PF3D7_1033200), PfRab1b GTPase (PF3D7_0512600), and the PVM protein S16 (Pfs16) (PF3D7_0406200) (**Table 1**). In other organisms, the Arf GTPase, Sec7, and Rab1b are involved in the transport from the *cis*-Golgi to the ER (Monetta et al., 2007). Another *Plasmodium*-specific candidate protein EBA181 is a ligand localized on the surface of merozoites and plays an important role in the entry of parasites into erythrocytes by binding to surface receptors on erythrocyte cell membranes (Gilberger et al., 2003). ETRAMP10.2 is expressed at an early point of the intraerythrocytic stage and is localized at the parasite periphery, which is assumed to be the PVM (Spielmann et al., 2003). The Pfs16 is expressed in gametocytes and is localized at the parasite periphery, similar to ETRAMP10.2 (Bruce et al., 1994). In this study, we focused on the functions of the PfArf and PfRab1b GTPases to elucidate the mechanism of intracellular transport mediated by PfRab5b. Studies on the other candidate proteins will be described elsewhere.

**Table 1:** Candidates for PfRab5b associated proteins.

Annotation	Gene ID	PlasmoDB ID	Molecular weight (kDa)	Normalized relative ratio against common peptidesPfRab5b-YFP-FLAG/PfRab5b-YFP (fold enrichment)
Rab5b	Q76NM7	PF3D7_1310600	23	72.8/1.16 (62.7)
Arf1	Q7KQL3	PF3D7_1020900	21	7.90/1.16 (6.80)
EBA181	Q8I2B4	PF3D7_0102500	181	7.90/1.16 (6.80)
Sec7	Q8IL42	PF3D7_1442900	405	11.4/2.32 (4.91)
ETRAMP10.2	Q8IJ76	PF3D7_1033200	39	5.27/1.16 (4.53)
Rab1b	Q7K6A8	PF3D7_0512600	23	4.39/1.16 (3.78)
Pfs16	Q6ZMA7	PF3D7_0406200	17	3.51/0 (N/A)

### PfArf1 and PfRab1b Colocalize With PfRab5b

In the *P. falciparum* genome, 11 Rab GTPase-encoding genes have been identified (Quevillon et al., 2003). Among them, PfRab1a and PfRab1b have been reported as two human Rab1 homologs (Quevillon et al., 2003). PfRab1a is localized to the ER and regulates trafficking from the ER to the apical organelles known as rhoptries (Morse et al., 2016). However, there are no reports for PfRab1b function and its subcellular localization. For the Sar/Arf family, the Sar1 homolog PfSar1 alone has been shown to localize the ER and define network membranes surrounding the parasite nuclei (Adisa et al., 2007). A BLASTP search revealed the presence of six Sar/Arf proteins in the *P. falciparum* 3D7 genome database (**Supplementary Figure 2A**). Amino acid sequencing showed that five of these proteins are Arf family GTPases (PF3D7_1020900, PF3D7-1034700, PF3D7_1442000, Pf3D7_0920500, Pf3D7_1316200), and one is a Sar1 homolog (PF3D7_0416800) based on the conserved effector sequence and the overall amino acid identities (**Supplementary Figure 2C**). PF3D7_1020900, which was obtained as PfRab5b interacting protein (**Table 1**), showed the highest identity (75%) to human Arf1 among the *Plasmodium* and human Arf families (**Supplementary Figure 2B**). Thus, the PfRab5b-associated protein candidate PF3D7_1020900 is a *Plasmodium* Arf1 homolog and was annotated as PfArf1 (GenBank Accession number, BR001667).

**Figure 1:**
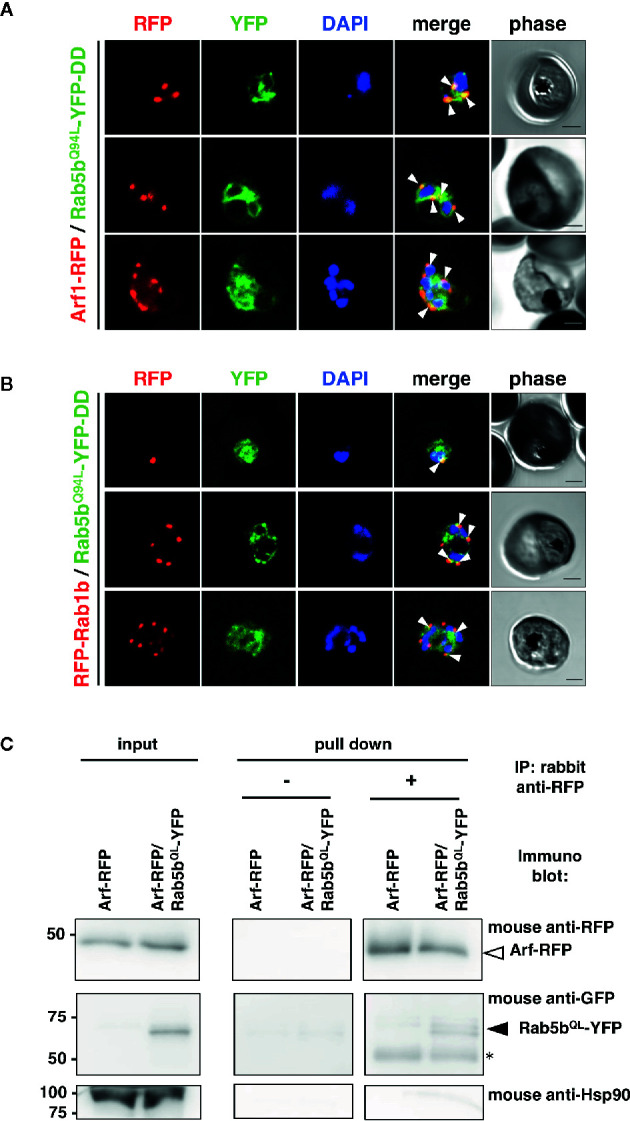
Association of PfRab5b and PfArf1 GTPases rather than PfRab1b in adjacent to the nucleus. Transformant parasites carrying the PfArf1-RFP (**A**, red****) or PfRab1b-RFP (**B**, red****) constructs with PfRab5b^Q94L^-YFP-DD (green) under the dual Pfef1α promoter were stabilized with Shld1, and were then used in the immunofluorescence assay. The fluorescence of RFP and YFP was captured. Arrowheads indicate the colocalization of PfArf1 and PfRab1b with PfRab5b^Q94L^. The nuclei were stained with DAPI (blue). Representative images showing mononuclear early trophozoite (upper), two nuclear late trophozoite (middle), and multinucleated early schizont (lower) stages are shown. White arrowheads indicate the colocalization of PfRab5b^Q94L^-YFP-DD and PfArf1-RFP or PfRab1b-RFP. The bars indicate 2 µm. **(C)** Reciprocal immuoprecipitation experiments of PfArf1-RFP *via* interaction with PfRab5b^Q94L^-YFP-DD. PfRab5b^Q94L^-YFP-DD and PfArf1-RFP double-expressing parasites were crosslinked with DSP as described in *Materials and Methods*, and immunoprecipitated with rabbit anti-RFP antibody (IP: +). Immunoprecipitated PfArf-RFP (a white arrowhead) and PfRab5b^Q94L^-YFP-DD (a black arrowhead) was visualized with mouse anti-RFP or anti-GFP antibodies, respectively. In the absence of rabbit anti-RFP antibody during immnoprecipitation (IP: −), neither PfArf-RFP nor PfRab5b^Q94L^-YFP-DD was detected. Anti-Hsp90 antibody was used as a negative control. Two 50 kDa bands in pull down fraction (an asterisk) were non specific recognition of secondary antibody against anti-rabbit IgG.

The intracellular colocalization of PfRab5b with PfArf1 or PfRab1b was demonstrated using double-expressing parasites. First, the constitutively active mutant PfRab5b^Q94L^ was fused with YFP and a DD (PfRab5b^Q94L^-YFP-DD) and expressed in a Shld1 ligand-dependent manner (Armstrong and Goldberg, 2007; Ebine et al., 2016). The Q-to-L substitution in the conserved GTP binding consensus domain impairs intrinsic GTPase activity which favor formation of the active GTP-bound form to small GTPases (Der et al., 1986: Stenmark et al., 1994). As conventional Rab GTPases are modified with C-terminal geranyl-geranylation (Joberty et al., 1993), PfRab1b was fused with the N-terminal RFP (RFP-PfRab1b). In contrast, Arf1 is modified with N-terminal myristoylation (Sewell and Kahn, 1988), and PfArf1 was fused with a C-terminus RFP fusion (PfArf1-RFP). PfRab5b^Q94L^-YFP-DD and RFP-PfRab1b, or PfArf1-RFP were placed under the control of the Pbef1α dual promoter and the constructs were transformed into parasites (**Supplementary Figure 1B**). Immunoblots using an anti-RFP antibody showed the 48 and 50 kDa bands of PfArf1-RFP and RFP-PfRab1b, respectively (**Supplementary Figure 3**). The anti-GFP antibody detected a 62 kDa band corresponding to PfRab5b^Q94L^-YFP-DD, indicating that the full length of fusion constructs were expressed (**Supplementary Figure 3**). In the mononuclear early trophozoite stage, 2 or 3 nuclear late trophozoite, and multinucleated early schizont stages, the RFP signals of PfArf1-RFP and RFP-PfRab1b were observed as juxtanuclear punctate structures, and these colocalized with the YFP fluorescence from PfRab5b^Q94L^-YFP-DD (**Figures 1A, B**). Previous report showed that PfRab5b localized near the parasite plasma membrane at the schizont stage (Ezougou et al., 2014). We additionally showed the localization of PfRab5b to the ER and PVM between ring and late schizont stages (Ebine et al., 2016). Thus we focus on the localization of PfArf1 and PfRab1b between early trophozoite and early schizont stages. PfRab5b^Q94L^-YFP-DD showed good colocalization with PfArf1-RFP (average Pearson’s correlation coefficient: *R* = 0.51 ± 0.088. n = 10 parasites). PfRab1b-RFP showed mild colocalization with PfRab5b^Q94L^-YFP-DD, as analyzed by YFP and RFP signals (average Pearson’s correlation coefficient: *R* = 0.34 ± 0.05, n = 10 parasites). This result indicated that PfArf1-RFP rather than PfRab1b-RFP closely associated to PfRab5b^Q94L^-YFP-DD (**Supplementary Figure 4**). The interaction of PfRab5b^Q94L^-YFP and RFP-PfArf1 was confirmed by reciprocal coimmunoprecipitation of RFP-PfArf1 and PfRab5b^Q94L^-YFP double-expressing parasites with anti-RFP antibody (**Figure 1C**). The immunoprecipitated PfRab5b^Q94L^-YFP was recognized as 62 kDa band using mouse anti-GFP antibody together with RFP-PfArf1 visualized with mouse anti-RFP antibody, while the negative control marker cytosolic protein Hsp90 was not detected in the sample. The interaction between PfRab5b^Q94L^-YFP and PfRab1b-RFP was not confirmed in this reciprocal coimmunoprecipitation (data not shown).

### PfArf1 and PfRab1b Were Localized in Different Subdomains of the ER and the *cis*-Golgi

**Figure 2:**
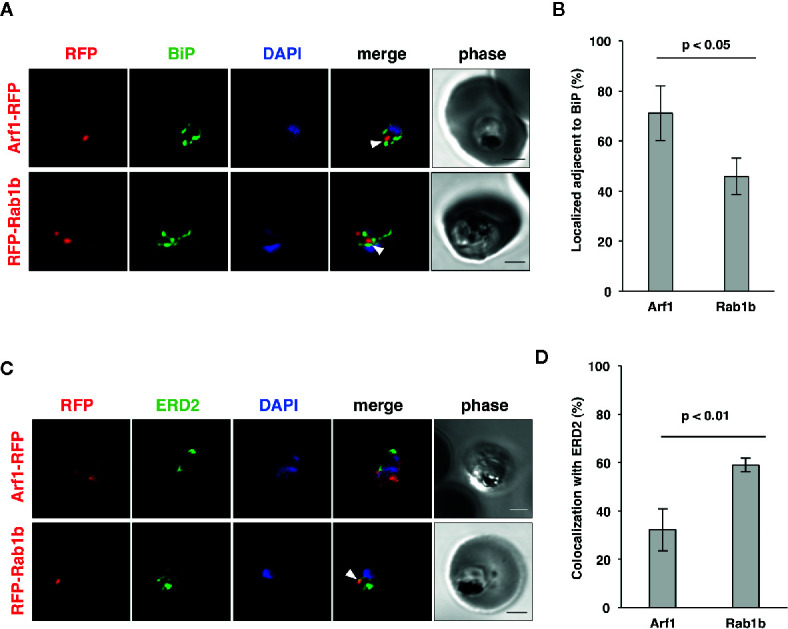
Localization of PfArf1 and PfRab1b in juxtaposition to the ER and the *cis*-Golgi. **(A)** Synchronized parasites, expressing PfArf1-RFP (upper, red) or RFP-PfRab1b (lower, red), were fixed at early trophozoite stage and subjected to the indirect immunofluorescence analysis with anti-PfBiP antibody (green) and DAPI (blue). The fluorescence of PfArf1-RFP and RFP-PfRab1b is shown. PfBiP was stained with an anti-PfBiP antibody. Both PfArf1-RFP and RFP-PfRab1b localized adjacent to the PfBiP signal (arrowheads). **(B)** Rate of colocalization of PfArf1-RFP and RFP-PfRab1b with PfBiP. The number of parasites that showed colocalization of the RFP and PfBiP signals was counted in 20–30 trophozoites from three independent experiments. Error bars indicate the standard deviations of three replicates. A test for statistical significance was performed using the Student *t*-test. **(C)** Indirect immunofluorescence analysis of the localization of PfArf1-RFP (upper, red), RFP-PfRab1b (lower, red), the *cis*-Golgi-marker PfERD2 (green), and DAPI (blue). The fluorescence from RFP-PfRab1b colocalized with the PfERD2 signal (arrowhead), but not with PfArf1-RFP. The bars indicate 2 µm. **(D)** Rate of colocalization of PfArf1-RFP and RFP-PfRab1b with PfERD2. The number of parasites that showed colocalization of the RFP and PfERD2 signals was counted in 20–30 trophozoites from three independent experiments. A test for statistical significance was performed using the Student *t*-test.

In an analysis conducted previously, we have shown that PfRab5b was localized adjacent to the ER (Ebine et al., 2016), which was labeled with the ER luminal chaperone PfBiP (Kumar et al., 1991; Kumar and Zheng, 1992). To examine the subcellular localization of PfArf1 and PfRab1b, which were colocalized with PfRab5b (**Figure 1B**), PfArf1-RFP, or RFP-PfRab1b expressing parasites (**Supplementary Figure 1C**) were stained with the anti-PfBiP antibody. Punctate structure signals for PfArf1-RFP and RFP-PfRab1b were closely localized with the PfBiP signals (**Figure 2A**). More than 70% of PfArf1-RFP expressing parasites showed colocalization of the PfArf1-RFP and PfBiP signals (71 ± 11%). This proportion is higher than that of RFP-PfRab1b and PfBiP in RFP-PfRab1b expressing parasites (46 ± 7%, *p* < 0.05) (**Figure 2B**). This result was unexpected because the Arf1 and Rab1 GTPases were previously reported to be localized and targeted to the *cis*-Golgi in most other organisms (Stearns et al., 1990; Moyer et al., 2001). Next, PfArf1-RFP and RFP-PfRab1b expressing parasites were stained with an anti-PfERD2 antibody, which stained the *Plasmodium* homolog of the *cis*-Golgi membrane protein ERD2 (Lewis and Pelham, 1990; Elmendorf and Haldar, 1993). Most of the PfArf1-RFP expressing parasites did not show colocalization of the PfArf1-RFP and PfERD2 signals (32 ± 9%) (**Figures 2C, D**). The ratio of PfRab1b colocalization with PfERD2 was increased to 59 ± 3% in PfRab1b-RFP expressing parasites (*p* < 0.05) (**Figures 2C, D**). These results indicate that both PfArf1 and PfRab1b simultaneously localize to the ER and *cis*-Golgi in this organism; however, the subcellular localization of fraction differed between PfArf1 and PfRab1b, as most of the PfArf1 was localized to the proximal region of the ER, and half of PfRab1b was individually localized to the ER and the *cis*-Golgi.

**Figure 3:**
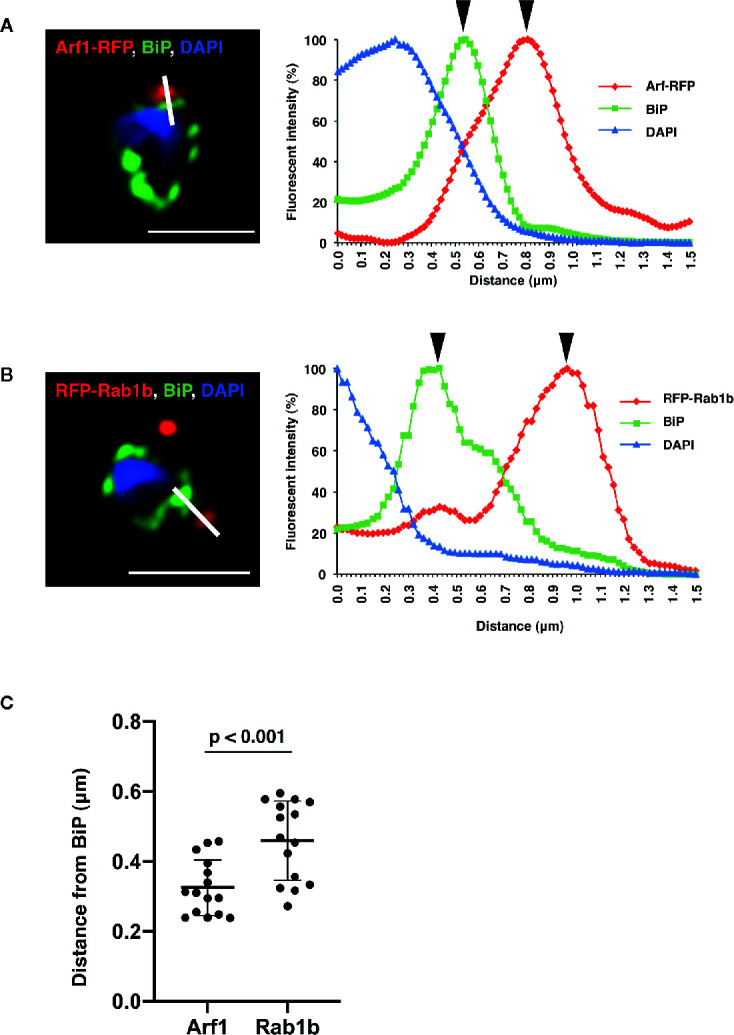
Super-resolution imaging showing the fine differences between PfBiP and PfArf1 or PfRab1b. Synchronized parasites, expressing PfArf1-RFP **(A,** red**)** or RFP-PfRab1 **(B**, red**)**, were sampled at early trophozoite stage and fixed and subjected indirect immunofluorescence analysis with the anti-PfBiP antibody (green) and DAPI (blue). Fluorescence intensities along the bold white lines are indicated in the graphs on the right. The fluorescence intensity was calculated as the percentage of the highest signal intensities. Black arrowheads depict the peaks of RFP and PfBiP intensities. The bars indicate 2 µm. **(C)** The smallest calculated distances between **(A, B)** in 15 independent parasites were plotted, and the average (bold bars) and standard deviation (thin bars) are indicated. A test for statistical significance was evaluated using the Student’s *t*-test.

Detailed analysis using super-resolution microscopy enabled the identification of the distinct subcellular localization of PfArf1 and PfRab1b on the ER, and whether both GTPases localize to the same subdomain or reside in distinct regions. The immunostained slides were processed with a super-resolution microscope LSM880 with Airyscan and processed with Zeiss Zen2 software, which provides a lateral resolution of 140 nm, to analyze the precise cellular locations of the proteins. Peak signal intensities of the most proximal staining between PfBiP and PfArf1-RFP (**Figure 3A**) or PfRab1b (**Figure 3B**) were calculated. The average distance from PfBiP was closer to PfArf1-RFP than to RFP-PfRab1 (PfArf1-RFP: 0.33 ± 0.08 µm *vs*. RFP-PfRab1 0.45 ± 0.11 µm, *p* < 0.001) (**Figure 3C**). These data indicate the presence of compartmentalization in the ER or ER adjacent novel membrane structures, and PfArf1 showed significant localization close to the PfBiP-positive ER rather than PfRab1b.

### PfArf1 and PfRab1b Are Involved in the Transport of the PEXEL-Positive Transmembrane Protein Rifin

**Figure 4:**
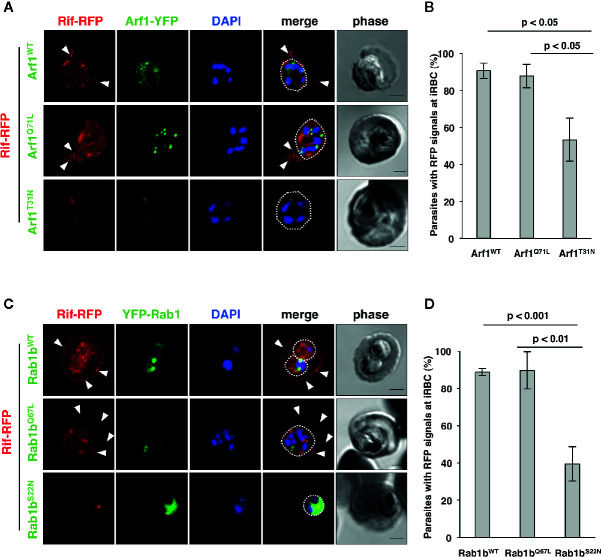
PfArf1 and PfRab1b regulated the export of PEXEL-positive Rifin to the erythrocyte cytoplasm. Parasites expressing Rifin-RFP and PfArf1-YFP-DD **(A, B)** or DD-YFP-PfRab1b **(C, D)** were examined *via* the immunofluorescence assay. Fluorescence signals from RFP (red), YFP (green), and DAPI (blue) are shown. Wild-type PfArf1 or PfRab1b (upper panels), active mutant PfArf1^Q71L^ or PfRab1b^Q67L^ (middle panels), and the inactive mutant PfArf1^T31N^ or PfRab1b^S22N^ (lower panels) are shown. White dotted lines indicate the parasite plasma membrane. The arrowheads indicate dot-like exported Rifin-RFP signals. The bars indicate 2 µm. The rate of parasites that showed a Rifin-RFP signal was detected in the erythrocyte cytoplasm in PfArf1-RFP **(B)** and RFP-PfRab1b **(D)** expressing cells are shown in graphs. Thirty individual early trophozoites and early schizonts were counted from three independent experiments. Infected RBCs, recognized by the DAPI and YFP signals under the microscope, were imaged by the laser microcopy, and then analyzed for the localization of RFP and whether Rifin-RFP was exported to the iRBC. The statistical significance was determined using the Student’s *t*-test.

Blood stage parasites export several proteins into the host erythrocyte cytosol and the PV (Sargeant et al., 2006; van Ooij et al., 2008). The PEXEL sequence is a five-residue motif in the downstream N-terminal signal peptide, and it has been detected in many exported proteins (Hiller et al., 2004; Marti et al., 2004). PEXEL-positive proteins have been suggested to pass through the classical ER/Golgi pathway (Akompong et al., 2002). However, the presence of several PNEPs indicates the existence of an alternative export pathway (Möskes et al., 2004; Thavayogarajah et al., 2015). We have previously shown that overexpression of PfRab5b did not disrupt the export of the PEXEL-positive transmembrane protein EVP1 to the iRBC cytosol (Ebine et al., 2016). To examine whether PfArf1 and PfRab1b are involved in trafficking of PEXEL-positive export proteins, we chose a Rifin variant PFA0745w, whose fusion construct with YFP was secreted into the erythrocyte cytosol (Marti et al., 2004). The N-terminal PEXEL domain and the C-terminal transmembrane region were fused with RFP (Rifin-RFP) and co-expressed with the PfArf1 and PfRab1b mutant constructs, whose expression was driven by the Shld1 ligand (**Supplementary Figure 1D**). In parasites expressing the PfArf1^WT^-YFP-DD and active mutant PfArf1^Q71L^-YFP-DD constructs, Rifin-RFP signals were detected in the iRBC plasma membrane and at the parasite periphery (PfArf1^WT^: 91 ± 4%, PfArf1^Q71L^: 88 ± 6%) (**Figures 4A, B** **)**. In contrast, the export of Rifin-RFP was reduced in the inactive PfArf1^T31N^-YFP-DD expressing parasites (53 ± 12%) (**Figures 4A, B** **)**, suggesting that PfArf1 is involved in PEXEL-positive Rifin transport. Similarly, the expression of wild-type DD-YFP-PfRab1b and the active mutant DD-YFP-PfRab1b^Q67L^ did not show differences for the export of Rifin-RFP (PfRab1b^WT^: 89 ± 2%, PfRab1b^Q67L^: 90 ± 10%), whereas the expression of the inactive mutant DD-YFP-PfRab1b^S22N^ reduced the export activity (39 ± 9%) (**Figures 4C, D** **)**.

### PfArf1, But Not PfRab1b, Regulates the Export of N-Acylated Adenylate Kinase 2 to the PVM

**Figure 5:**
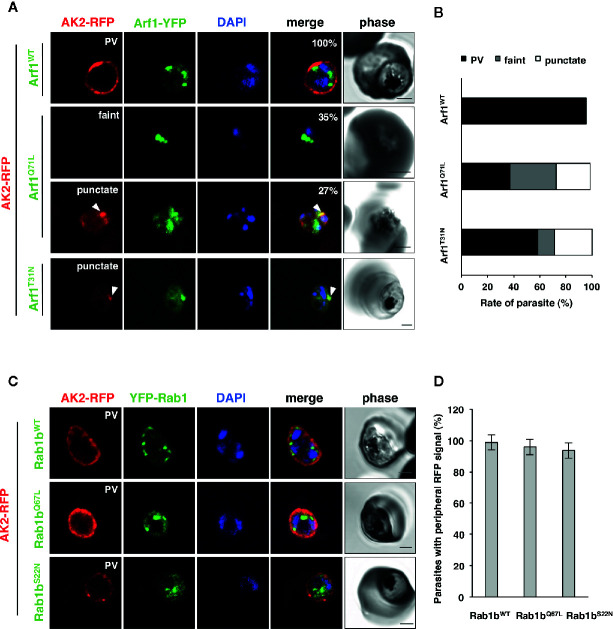
The specific role of PfArf1 in the transport of N-acylated PfAK2 to the PVM. Parasites expressing PfAK2-RFP and PfArf1-YFP-DD **(A, B)** or DD-YFP-PfRab1b **(C, D)** were examined using the immunofluorescence assay. Fluorescence signals from RFP (red), YFP (green), and DAPI (blue) are shown. Wild-type PfArf1 or PfRab1b (upper panels), the active mutant PfArf1^Q71L^ or PfRab1b^Q67L^ (middle panels), and the inactive mutant PfArf1^T31N^ or PfRab1b^S22N^ (lower panels) are shown. Representative images for PfAK2-RFP are shown and are divided into three patterns: peripheral PVM staining (PVM), faint signal (faint), and dot-like punctate signal within the parasites (punctate). The bars indicate 2 µm. The parasites that showed a signal for PfAK2-RFP were classified into three patterns based on the PfArf1-RFP expressing cells **(B)** and then shown in bar graph. The rate of parasites showing peripheral staining for PfAK2-RFP in RFP-PfRab1b expressing cells **(D)**. Thirty individual early trophozoites and early schizonts were counted from three independent experiments. Infected RBCs, recognized by the DAPI and YFP signals under the microscope, were imaged by the laser microcopy, and then analyzed for the localization of RFP signal as PV, faint, and punctate. The statistical significance was determined using the Student’s *t*-test.

Adenylate kinase 2 (PfAK2) is an N-terminal myristoylated and palmitoylated protein, which lacks the signal peptide and a transmembrane domain. It localizes to the parasite plasma membrane face to the PV (Thavayogarajah et al., 2015; Ebine et al., 2016), suggesting that PfAK2 was not transported through the classical ER/Golgi-dependent pathway. We have previously reported that overexpression of PfRab5b-YFP-DD disrupted the transport of PfAK2-RFP to the PVM (Ebine et al., 2016). Overexpression of PfRab5b-YFP-DD altered the peripheral staining of PfAK2-RFP in the parasite cytoplasmic staining pattern, indicating that PfRab5b might be involved in the transport of PfAK2 (Ebine et al., 2016). Therefore, we examined whether the overexpression of PfArf1 perturbs the transport of PfAK2-RFP to the PVM. Parasites that double-expressed PfAK2-RFP and PfArf1^WT^, or the constitutively active PfArf1^Q71L^ or inactive PfArf1^T31N^ mutants (**Figure 1A**), whose expression is driven by the Shld1 ligand were established (**Supplementary Figure 1D**). The PfArf1^Q71L^ and PfArf1^T31N^ mutants are corresponding to human Arf1^Q71L^ and Arf1^T31N^, respectively (Dascher and Balch, 1994: Teal et al., 1994). Expression of PfArf1^Q71L^ or PfArf1^T31N^ mutants for 48 h did not show growth defect (**Supplementary Figure 5**). In the PfArf1^WT^-YFP-DD expressing parasite, all PfAK2-RFP signals were localized at the parasite periphery, indicating a typical PVM staining pattern (**Figure 5A**). In contrast, the active mutant PfArf1^Q71L^-YFP-DD expressing parasites reduced PfAK2-RFP targeting to the PVM (38 ± 6.7%). Several parasites showed a faint RFP signal and a punctate RFP signal within the parasite cytoplasm (faint: 35 ± 7.1%, punctate: 27 ± 7.7%, respectively) (**Figures 5A, B**). In the inactive PfArf1^T31N^-YFP-DD expressing parasite, the transport of PfAK2-RFP was reduced to 58 ± 6.8%, and further, 13 ± 7.2% and 29 ± 11% of the parasites showed a faint RFP signal and a punctate pattern in the cytoplasm, respectively (**Figures 5A, B**). The faint signal of PfAK2-RFP was more abundant in PfArf1^Q71L^-YFP-DD than in PfArf1^T31N-^YFP-DD (*p* < 0.05). These results indicate that PfArf1 is directly involved in the trafficking of PfAK2 to the PVM. The specific role of PfArf1 in the transport of PfAK2 was highlighted by the co-expression of PfRab1b mutants (**Figure 5C**). In the co-expression with the wild-type DD-YFP-PfRab1b, active DD-YFP-PfRab1b^Q67L^, or the inactive DD-YFP-PfRab1b^S22N^ constructs, which corresponds human Rab1b^Q67L^ and Rab1b^S22N^, respectively (Tisdale et al., 1992), the transport of PfAK2-RFP was not inhibited and all parasites showed a peripheral pattern for their expression (PfRab1b^WT^: 99 ± 2%, PfRab1b^Q67L^: 96 ± 4%, PfRab1b^S22N^: 94 ± 3%) (**Figures 5C, D**). Expression of Rab1b^Q67L^ and Rab1b^S22N^ mutants for 48 h did not show growth defect (**Supplementary Figure 5**). These results suggest that PfArf1 is extensively involved in the transport of the N-acylated protein PfAK2 to the PVM.

## Discussion

### Isolation of PfArf1 and PfRab1b as PfRab5b Associated Proteins

In this study, we demonstrated the isolation of PfRab5b proteins using a coimmunoprecipitation approach. We identified two GTPases, PfRab1b and the human Arf1 homolog PfArf1, that colocalized with PfRab5b and were associated with the ER marker PfBiP in erythrocytic stage parasites. Further, we demonstrated that PfArf1 and PfRab1b are closely located near the ER, but the precise localization differed as shown by super-resolution microscopy, suggesting that the ER-Golgi interface in *Plasmodium* is highly compartmentalized. Additionally, we provide direct cell biological evidence that PfArf1 and PfRab1b are involved in different types of cargo selection. PfArf1 regulates the transport of N-acylated PfAK2 to the PVM, whereas PfRab1b controls the trafficking of the PEXEL-positive exported protein Rifin in the erythrocyte cytosol. Thus, PfRab5b and its associated GTPases are involved in the sorting of several families of exported proteins in the different ER subdomains.

Rab GTPase recycles GTP-bound active and GDP-bound inactive forms (Hutagalung and Novick, 2011). To immunoprecipitate efficiently, we used DSP cross-linker, whose spacer arm length 12.0 Å, before the coimmunoprecipitation to fix the PfRab5b-nucleotide bound state. Such approach, the use of cross-linker before coimmunoprecipitation, identifies many (> 100) proteins in LC-ToF MS/MS (Watanabe et al., 2020). Our approach also identified 677 proteins and PfArf1 was listed as highly enriched proteins among 677 proteins (**Supplementary Table 2**). Contrary, a casein kinase CK1, which is identified as PfRab5b binding protein by bioinformatic technique (Rached et al., 2012), was not enriched in our approach (Accession Number, C6S3F7-1, **Supplementary Table 2**). Colocalization of PfRab5b and CK1 at parasite periphery was reported in schizont stage (Ezougou et al., 2014). These observations suggested that PfRab5b and CK1 may localized to different membrane subdomain on PVM in schizont stage. Further biological and biochemical studies are needed to prove direct and indirect interaction between PfRab5b and effector proteins.

### PfArf1 Exports the N-Myristoylated Protein PfAK2 to the PVM

The regulatory mechanisms underlying the trafficking of acylated proteins are not clearly understood in *Plasmodium* and other organisms. Dual acylated proteins are first myristoylated at the ER membrane by N-myristoyl transferase after palmitoylation by a palmitoyltransferase, and the acylated proteins are then trafficked to the apical organelle and parasite plasma membrane surface (Cabrera et al., 2012). For other proteins, such as the *Plasmodium falciparum* calcium-dependent protein kinase 1 (PfCDPK1) and the *Drosophila* transgultaminase A (TG-A), the dual acylated proteins are packed into multivesicular bodies and are subsequently exported to the apical organelle or extracellular space *via* the unconventional ER-Golgi-independent pathway (Möskes et al., 2004; Shibata et al., 2017). We have previously shown that the transport of N-myristoyl PfAK2 to the PVM was inhibited by the overexpression of PfRab5b, and that PfAK2 accumulated in the punctate structure within the parasite cytoplasm together with PfRab5b, indicating that PfRab5b and PfAK2 were included in the internal vesicle of the multivesicular body and were then transported to the PVM (Ebine et al., 2016). In this study, we have shown that PfArf1, but not PfRab1b, is involved in the regulation of PfAK2 (**Figures 4** and **5**). Our results indicated that the expression of active or inactive PfArf1 mutants inhibited PfAK2 transport to PVM (**Figure 5A**), whereas the expression of active or inactive PfRab1b mutants had no effect (**Figure 5C**). These results suggest that GTP hydrolysis by PfArf1 is required for proper PfAK2 recognition and further transport. For mammalian Arf1, it has been biochemically demonstrated that GTP hydrolysis by human Arf1 promotes the selective cargo selection from the Golgi membrane, and the concentration of cargo proteins into the COPI-coated vesicles (Lanoix et al., 1999). This observation indicated that the role of GTP hydrolysis in cargo selection is similar between PfArf1 and the human Arf1.

### Presence of Subdomains Near the ER and the Sequential Roles of PfArf1 and PfRab1b in Cargo Selection

Both PfArf1 and PfRab1b are localized close to the ER as punctate structures in the early erythrocytic stage (**Figure 1B**). The punctate structure near the ER was observed in other proteins, such as the COPII coat components PfSec13 and PfSec24 (Lee et al., 2008; Struck et al., 2008) and in another PfRab1 isotype PfRab1a (Morse et al., 2016). Previously, we have shown that PfRab5b did not colocalize with the COPII component PfSec13 (Struck et al., 2008; Ebine et al., 2016), suggesting that PfArf1 and PfRab1b might be localized in different domains from the COPII vesicle budding site, the ERES. In this study, the results indicate that the PfArf1 signal was not completely consistent with that for PfRab1b and was localized to different membrane subdomains around the ER (**Figure 3**). These findings suggest that the previously reported COPII component, PfArf1, and PfRab1b might be localized to an independent subdomain and may have different roles in the transport of cargo proteins and cargo selection from the ER. PfRab1b was involved in the transport of the PEXEL-positive export protein, Rifin, and the expression of the inactive PfRab1b mutant showed decreased activity of Rifin export to erythrocyte cytosol (**Figures 4C, D** **)**. In contrast, the expression of the active PfRab1b mutant did not inhibit Rifin export, indicating that the GTP-bound state of PfRab1b may be necessary for the proper functioning of PfRab1b. A similar observation was reported in mammalian Rab5 during endosome fusion, where it was demonstrated that the GTP-bound mutant form showed the same effect as the wild-type Rab (Barbieri et al., 1996).

**Figure 6:**
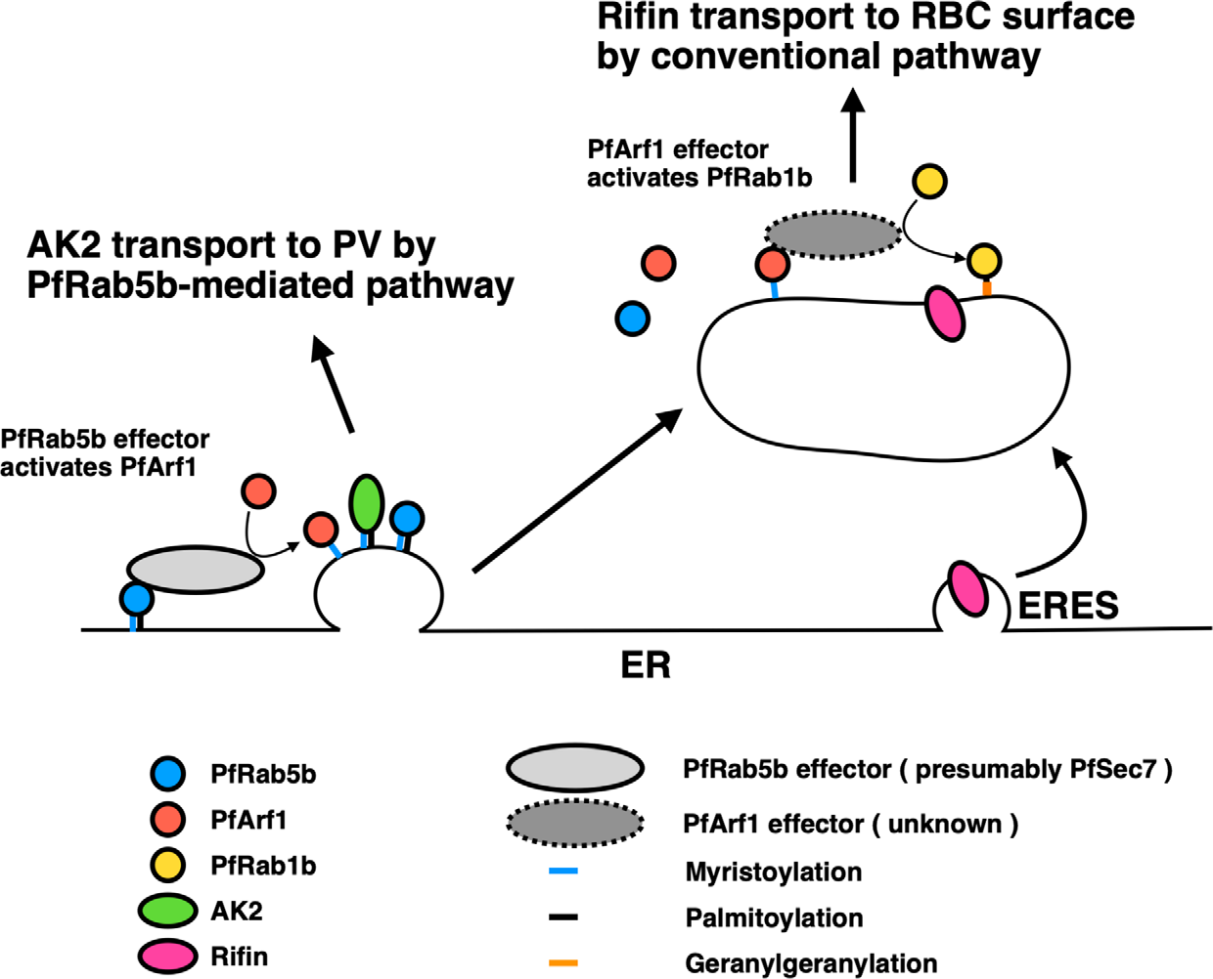
A proposed model for the two pathways regulated by PfArf1 and PfRab1b. PfRab5b (blue circle) recruited PfArf1 (red circle) probably through the binding to PfArf1 activating protein (light grey ellipse) on the ER membrane (**Figure 1**). Membrane targeted PfArf1 selected the N-acylated AK2 and sorted into the pathway for the PV (**Figure 5**). According to the partial involvement of PfArf1-positive membrane for the selection of PEXEL-positive Rifin (**Figure 4**), and PfRab1b (yellow circle) localized to distant membrane from the ER (**Figure 3**), PfArf1-positive membrane might be matured into PfRab1b-positive compartment. PfRab1b-positive compartment selected and further transport the PEXEL-positive Rifin to the RBC surface. ER, endoplasmic reticulum; ERES, endoplasmic reticulum exit site; PV, parasitophorous vacuole; RBC, red blood cell.

Interestingly, the export of Rifin was also decreased by the expression of inactive mutants of PfArf1, but not the active GTP-fixed mutant and the wild-type PfArf1, which cycles GTP- and GDP-bound states (**Figures 4A, B** **)**. This result suggests that the GTP-bound state of PfArf1, but not GDP-fixed mutant, is necessary for the export of Rifin, or for the correct activity of PfRab1b. Although not included in this study, it is notable that a Sec7 domain containing protein was listed as a candidate for the PfRab5b binding protein using mass analysis (**Table 1**). The 200 amino acid residue Sec7 domain is conserved among eukaryote and has the guanine nucleotide exchange activity toward Arf (Arf GEF) (Cherifils et al., 1998). Sec7 binds GDP-bound wild-type Arf GTPase and promotes the exchange of GDP for GTP. In other organisms, Sec7 is involved in the “Rab cascade”: activated GTPase triggers the recruitment of GEF for the downstream GTPase, and thus a series of GTPase activations is feasible (Barr, 2013). For example, in *Saccharomyces cerevisiae*, Ypt1 (in yeast Rab1) and the Arf-like GTPase Arl1 recruit Sec7 to the Golgi membrane, and subsequently Arf1 is activated by the GEF activity of membrane-localized Sec7, following the stimulation of Ypt31 (yeast Rab11) on the trans-Golgi membrane for cargo sorting to secretory vesicles (McDonold and Fromme, 2014). Thus, we generated a sequential hypothetical model of PfArf1 and PfRab1b adjacent to the ER that is presented in **Figure 6**. Membrane-localized PfRab5b may recruit PfSec7 to exchange GDP for GTP of PfArf1 in the adjacent ER subdomain, where the N-myristoylated protein PfAK2 is selected and packed into the pathway destined for the PVM. This is based on the finding that colocalization of the active mutant of PfRab5b and PfArf1 (**Figure 1**), and wild-type PfArf1 is necessary for the transport of PfAK2 (**Figure 5A**). It has been shown that *Plasmodium* PfArf1 possesses GTPase activity *in vitro* (Stafford et al., 1996) and that PfSec7 accelerated the nucleotide exchange activity against PfArf1 (Baumgartner et al., 2001). Thus, the PfArf1 may colocalize with PfSec7 adjacent to the ER. The role of PfSec7 in intracellular traffic remains elusive, and further studies are needed to unravel the regulation of PfAK2 transport together with PfArf1. Subsequently, the PfArf1 positive membrane matures and recruits PfRab1b at the membrane. This model is based on our results that indicate that PfRab1b localizes to the different subdomains of PfArf1 that are adjacent to the ER (**Figures 1B** and **3**), and that the sequential recruitment of different GTPases to the membrane occurs *via* Sec7 (McDonold and Fromme, 2014). In this hypothesis, PfSec7 could not recruit on the membrane in GDP-fixed PfArf1 mutant and may showed the defect in the function of downstream PfRab1b, suggesting expression of inactive mutant of PfArf1 showed the reduction of the export of Rifin (**Figures 4A, B** **)**. The Rab1 positive membrane subdomain near the ER is found in mammalian tubulovesicular membrane clusters of the ER-Golgi intermediate compartment (ERGIC) (Appenzeller-Herzog and Hauri, 2006). ERGIC clusters lie close to the COPII-positive ERES. Transport from the ER to the ERGIC is controlled by COPII coat vesicles, and Rab1 is involved in membrane tethering at the ERGIC in anterograde transport (Allan et al., 2000). Sorting in the ERGIC involves another protein coat of COPI and the Arf family (Goldberg, 2000). Thus, ERGIC is a sorting and recycling platform for the transport between the ER and Golgi in mammalian cells. In *Plasmodium*, the PfRab1b-positive compartment was involved in the export of the PEXEL-positive protein Rifin (**Figure 4**) and this suggests the existence of a novel sorting compartment for PEXEL-positive cargo proteins. The model shown in **Figure 6** indicates the segregation of the subdomain for sorting of different cargo proteins. Conversely, the expression of active and inactive PfRab1b mutants did not affect the export of PfAK2 (**Figure 5**).

### Transport to the Golgi and the Presence of a Few Parts of PfArf1 on the PfERD2-Positive Golgi Membrane

In most mammalian secretory cells, electron microscopy has revealed the presence of ribosome-coated rough ER and partly smooth surfaced structures in the vicinity of the Golgi complex (Saraste and Kuismanen, 1992). Golgi is displayed as stacks of flattened cisternae, which are often laterally linked into a ribbon-like structure (Zhang and Wang, 2016). In contrast, the ER and Golgi of malarial parasites are not well characterized and are reported to be loosely associated vesicles (Aikawa, 1971). To overcome the difficulty in the visualization of ER and the Golgi, we used super-resolution fluorescence microscopy (**Figure 3**) and quantified the number of colocalized parasites (**Figures 2**–**5**). In this study, about 70% of parasites showed colocalization of PfArf1 and the ER marker PfBiP, and 30% showed colocalization with the *cis*-Golgi marker PfERD2 (**Figure 2**). These results may explain the following two possibilities. One; PfArf1 is a Golgi resident which is involved in retrograde trafficking from the Golgi. This model is based on reports from mammalian and yeast models, where Arf1 is involved in different steps of cargo sorting together with specific effector or Sec7 containing proteins such as retrograde traffic from the *cis*-Golgi to the ER, or the secretory pathway from the *trans*-Golgi (Donaldson and Jackson, 2011). As it is not yet characterized, the Golgi resident PfArf1 may be involved in retrograde trafficking to the ER because the homolog of ERD2 is present and localized to the Golgi in *Plasmodium* (Elmendorf and Haldar, 1993). ERD2 retrieves the conserved C-terminal tetrapeptide sequence HDEL-containing ER luminal proteins from the *cis*-Golgi in yeast and mammalian cells (Hsu et al., 1992; Townsley et al., 1994). A second possibility is the presence of the Golgi subdomain in *Plasmodium*. The *Plasmodium* ERD2 was previously reported to colocalize with the Golgi re-assembly stacking protein (GRASP) (Struck et al., 2005), and PfArf1 was shown to be colocalized with GRASP in the early trophozoite stage (Thavayogarajah et al., 2015). Therefore, it appears that PfArf1, GRASP, and PfERD2 are colocalized in the same Golgi membrane. However, mammalian GRASP55 is present in the *medial*/*trans*-cisternae of Golgi stacks as shown by cryo-immunoelectron microscopy (Langreth et al., 1978). Thus, this may be the reason for the Golgi-stacked protein GRASP and HDEL-receptor ERD2 to colocalize in the same Golgi subdomain in *Plasmodium*.

## Conclusion and Future Perspectives

In conclusion, our results show that PfArf1 mediates the transport of N-myristoylated PfAK2 from the adjacent ER, and PfRab1b, which localizes differently than PfArf1, is involved in the export of PEXEL-positive Rifin to the erythrocyte cytosol. Currently, the mechanism how PfArf1 recognize cargo protein at the ER subdomain and sort to the pathway for the PVM, remains elusive. In other cases, there are very few reports on trafficking of dual acylated protein *via* a multivesicular body (Möskes et al., 2004; Shibata et al., 2017). In mammalian cells, myristoylated and palmitoylated GFP localized to the membrane subdomain enriched with cholesterol and ganglioside at the plasma membrane (McCabe and Berthiaume, 2001). It may plausible that PfArf1 together with PfRab5b and unidentified effector proteins organize the acylated cargo-recognition subdomain at the ER lipid subdomain. Consideration of the two facts that PfRab5b is essential for the growth (Ezougou et al., 2014; Ebine et al., 2016) and N-myristoyl transferase is a promising drug target for malaria (Schlott et al., 2018), suggests that elucidation of further molecular mechanism on PfArf1 and its regulatory proteins may help the plasmodium biology as well as pathogenesis.

## Data Availability Statement

The data sets presented in this study can be found in online repositories. The names of the repository/repositories and accession number(s) can be found in the article/**Supplementary Material**.

## Author Contributions

IT, TH, and YS-N conceived the study. IT, TH, TM, NS, SI, and YS-N designed and performed experiments and data analysis. IT, TM, TA, and YS-N drafted the paper. TA, KN, and TN participated in data analysis and edited the paper. YS-N acquired grants. All authors contributed to the article and approved the submitted version.

## Funding

This research was funded by Grants-in-Aid for Scientific Research (C) (JP19K07531 to YS-N) from the Ministry of Education, Culture, Sports, Science and Technology (MEXT), and a grant from The Naito Foundation to YS-N.

## Conflict of Interest

The authors declare that the research was conducted in the absence of any commercial or financial relationships that could be construed as a potential conflict of interest.

## Supplementary Material

Supplementary Table 1 List of oligonucleotide sequences used for plasmid construction.

Supplementary Table 2 List of proteins detected from immunoprecipitated samples prepared from PfRab5b-YFP-FLAG and PfRab5b-YFP expressing parasites using anti-FLAG antibody. The most enriched proteins were shown in red and listed in **Table 1**.

Supplementary Figure 1 Schematic structure of the constructs used in this study. **(A)** Episomal vector pCHD43(II) (Sakura et al., 2013; Ebine et al., 2016) was modulated to express a fusion fragment of PfRab5b-YFP-FLAG or (upper) or PfRab5b- YFP (lower). **(B)** The artificial centromere plasmid PfCenV-ef1-double was inserted two fragments in NcoI and NdeI sites, whose expression is regulated under the *Plasmodium berghei* elongation factor 1 (Pbef1α). In NcoI site, PfRab5b-YFP-DD was inserted and RFP-PfArf1 (upper) or PfRab1b-RFP (lower) was inserted in the NdeI site. **(C)** RFP-PfArf1 (upper) or PfRab1b-RFP (lower) was inserted in the NdeI site of the centromere plasmid for the single expression. **(D)** To double express GTPase (PfArf1 and PfRab1b) and cargo proteins (PfAK2 and Rifin), PfArf1-YFP-DD (upper, left) or DD-YFP-PfRab1b (lower, left) fragment was inserted into episomal plasmid pCHD43(II), whose hDHFR cassette was replaced with a blasticidin S-resistance cassette (Ebine et al., 2016). Cargo proteins, PfAK2-RFP (upper, right) or Rifin-RFP (lower, right) fusion constructs, were inserted into PfCenV-ef1-double of NdeI site. Each GTPase and cargo protein construct was transfected into the parasite, and simultaneously selected with WR99210 and blasticidin S. proCRT, the constitutive *P. falciparum* chloroquine resistance transporter promoter: PbDT, *Plasmodium berghei* dihydrofolate reductase terminator proPfCAM, *P. falciparum* calmodulin promoter, hDHFR, human dihydrofolate reductase: PfHRPII, *P. falciparum* histidine-rich protein II termiator: rep20, element to improve episomal segregation at mitosis: PfCenV, the centromere of chromosome 5: BSD, blasticidin S.

Supplementary Figure 2 Pf3D7_1020900 is a mammalian homolog of Arf1. **(A)** Amino acid percentage identity matrix of the six *Plasmodium* Sar/Arf familiy of GTPases and human Arf1 and Sar1. *Plasmodium* protein sequences for the Sar/Arf family of GTPases were retrieved from the PlasmoDB genome database (release 46, Nov 2019) using the Pathema Bioinformatics Resource Center (https://plasmodb.org/plasmo/) (Collaborative, 2001). *Plasmodium* Sar/Arf homologs were retrieved *via* the BLASTP algorithm using the human Arf1 and Sar1 GTPases as queries in the *P. falciparum* 3D7 database. **(B)** Sequence alignment of *Homo sapiens* Arf1 and Sar1 and six *Plasmodium* Sar1/Arf families of proteins. Amino acid sequences were aligned using Clustal Omega (EMBL-EBI, Welcome Genome Campus). The N-terminus myristoylation site with a glycine residue specific for the Arf family and the GTP-binding consensus boxes are indicated in blue and red boxes, respectively (Pereira-Leal and Seabra, 2000). The effector regions of the Arf/Arf-like (Arl) and Sar1 families are indicated in light blue and magenta, respectively. Note that PF3D7_1442000 and PF3D7_1316200 lack an N-terminal glycine residue. PF3D7_0920500 and PF3D7_1316200 are missing the second GTP-binding box. Two conserved residues, that are substituted to create constitutively active and inactive mutants, are indicated by black and white arrowheads, respectively. **(C)** Amino acid percentage identity matrix of Pf3D7_1020900 and the five human Arf1.

Supplementary Figure 3 Immunoblots showing the expression of full length of PfArf1-RFP, and RFP-PfRab1b. Parasite lysates expressing PfRab5b^Q94L^-YFP-DD and Arf1-RFP or RFP-PfRab1b were subjected to immunoblot analysis using anti-RFP and anti-GFP antibodies. Anti-Hsp90 antibody was used as a loading control. Mock is the non-transformant parasite.

Supplementary Figure 4 Pearson’s correlation coefficient between PfRab5b^Q94L^-YFP-DD and PfArf1-RFP or RFP-PfRab1b. The fluorescence intensities of the merged YFP and RFP signals were analyzed using Fiji-Image J software to generate Pearson correlation coefficients. The scatter plot represents Pearson correlation coefficients values from ten independent parasites, which are obtained from three independent transfectants. The statistical significance was determined using Student’s t-test.

Supplementary Figure 5 Expressions of PfArf1 or PfRab1b mutants showed no effect on parasites growth. The population doubling time of non-transformant MS822 line, PfArf1 and PfRab1b mutants were measured for 2 days cultivation from three independent assays. The average (bar graph) and the standard deviation (thin bar) are indicated. Mock was cultured without drugs and PfArf1, PfRab1b mutants were cultured with BSD. Except from MS822 line, transformed parasite were cultured in the presence of 2.5 µg/ml of BSD and the expressions of PfArf1 and PfRab1b mutants were stabilized by 0.5 µM Shld1. A statistical significance was evaluated using Student’s *t*-test.
