# Supplementary figures and images for: Rab5b-Associated Arf1 GTPase Regulates Export of N-Myristoylated Adenylate Kinase 2 From the Endoplasmic Reticulum in Plasmodium falciparum
Source: Front Cell Infect Microbiol. 2021 Feb 2;10:610200. doi: 10.3389/fcimb.2020.610200 (PMC7884776; doi:10.3389/fcimb.2020.610200)

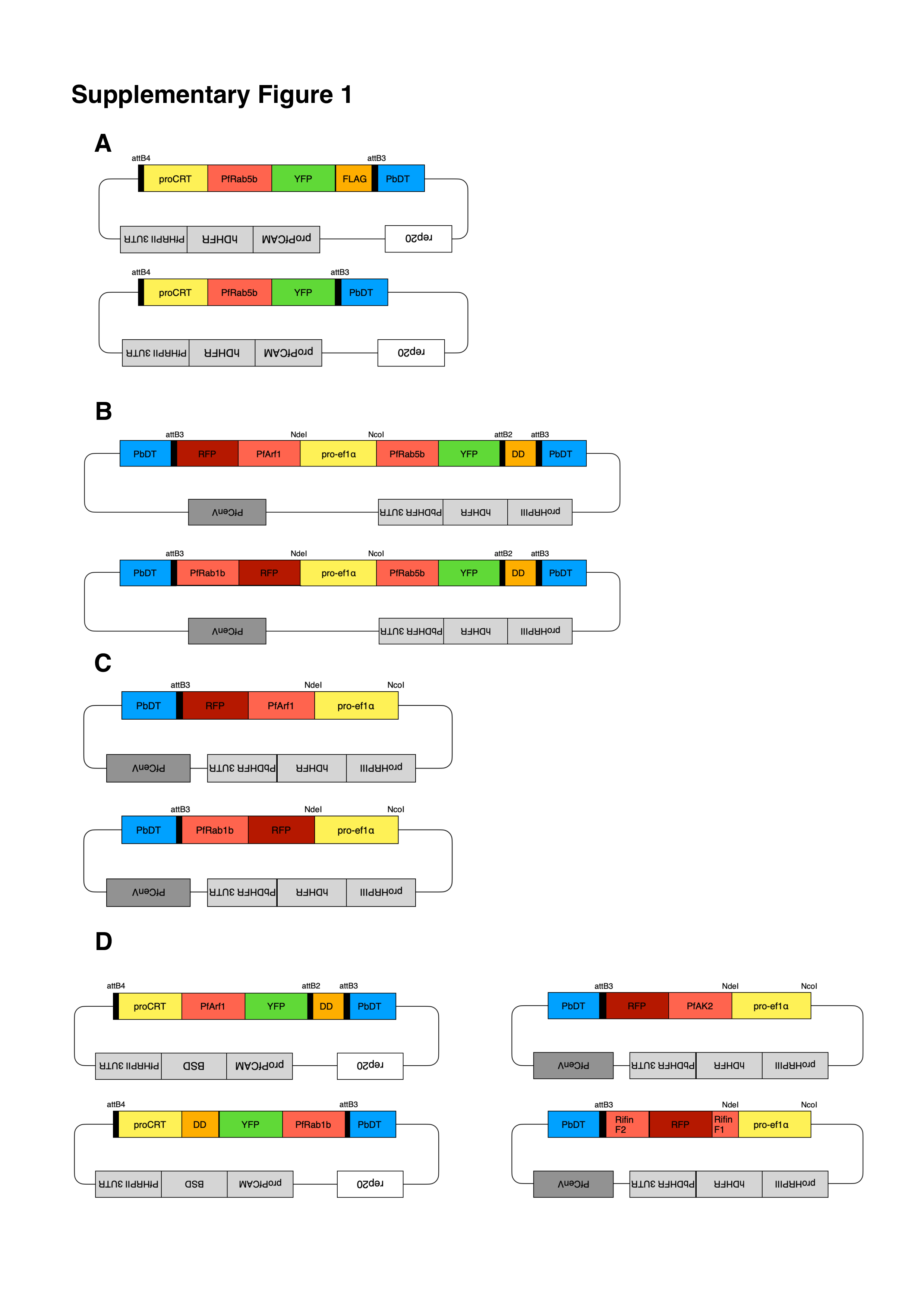

Supplement: Supplementary Figure 1 — Schematic structure of the constructs used in this study. (A) Episomal vector pCHD43(II) (Sakura et al., 2013; Ebine et al., 2016) was modulated to express a fusion fragment of PfRab5b-YFP-FLAG or (upper) or PfRab5b- YFP (lower). (B) The artificial centromere plasmid PfCenV-ef1-double was inserted two fragments in NcoI and NdeI sites, whose expression is regulated under the Plasmodium berghei elongation factor 1 (Pbef1α). In NcoI site, PfRab5b-YFP-DD was inserted and RFP-PfArf1 (upper) or PfRab1b-RFP (lower) was inserted in the NdeI site. (C) RFP-PfArf1 (upper) or PfRab1b-RFP (lower) was inserted in the NdeI site of the centromere plasmid for the single expression. (D) To double express GTPase (PfArf1 and PfRab1b) and cargo proteins (PfAK2 and Rifin), PfArf1-YFP-DD (upper, left) or DD-YFP-PfRab1b (lower, left) fragment was inserted into episomal plasmid pCHD43(II), whose hDHFR cassette was replaced with a blasticidin S-resistance cassette (Ebine et al., 2016). Cargo proteins, PfAK2-RFP (upper, right) or Rifin-RFP (lower, right) fusion constructs, were inserted into PfCenV-ef1-double of NdeI site. Each GTPase and cargo protein construct was transfected into the parasite, and simultaneously selected with WR99210 and blasticidin S. proCRT, the constitutive P. falciparum chloroquine resistance transporter promoter: PbDT, Plasmodium berghei dihydrofolate reductase terminator proPfCAM, P. falciparum calmodulin promoter, hDHFR, human dihydrofolate reductase: PfHRPII, P. falciparum histidine-rich protein II termiator: rep20, element to improve episomal segregation at mitosis: PfCenV, the centromere of chromosome 5: BSD, blasticidin S. [file Image_1.tiff]

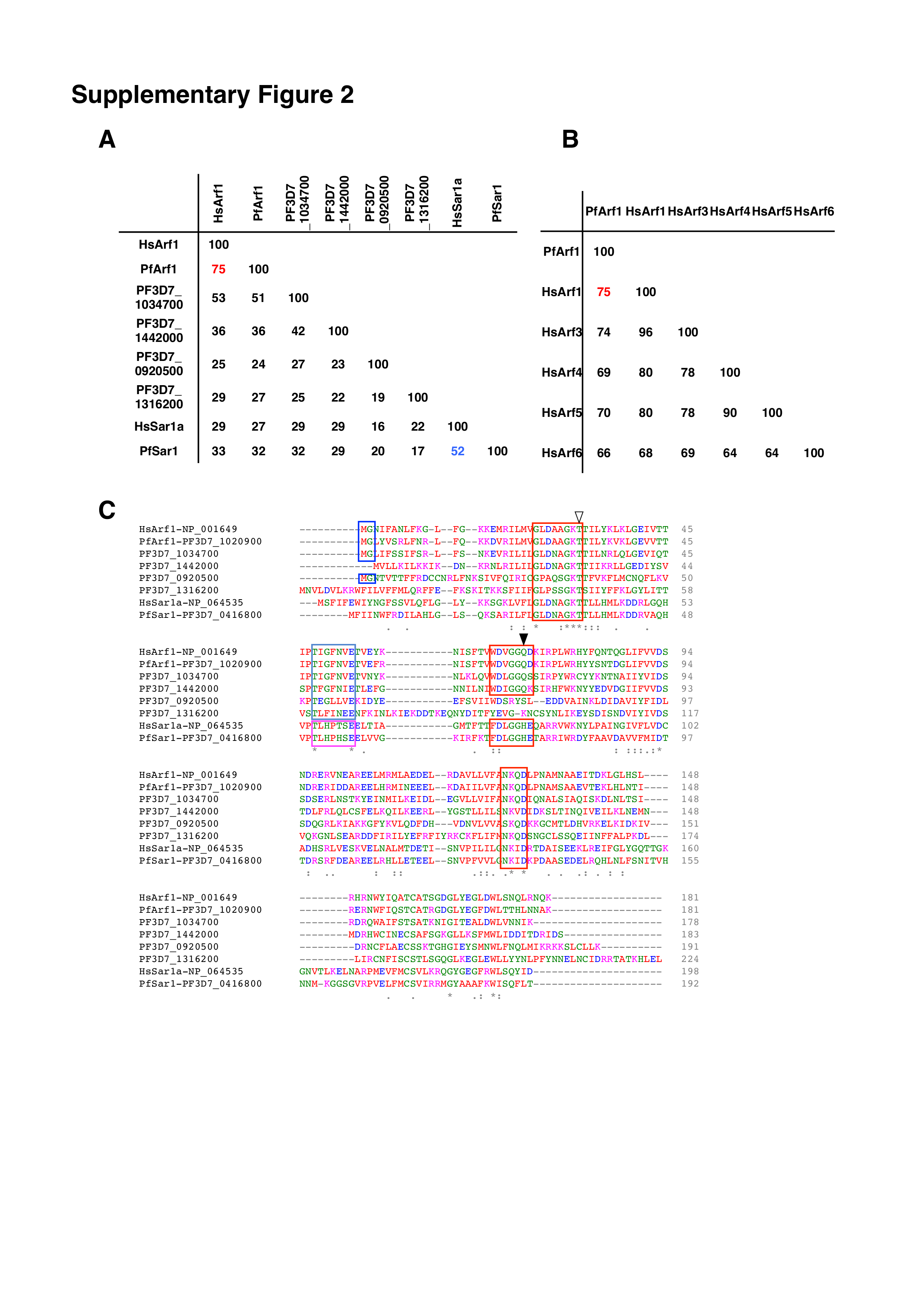

Supplement: Supplementary Figure 2 — Pf3D7_1020900 is a mammalian homolog of Arf1. (A) Amino acid percentage identity matrix of the six Plasmodium Sar/Arf familiy of GTPases and human Arf1 and Sar1. Plasmodium protein sequences for the Sar/Arf family of GTPases were retrieved from the PlasmoDB genome database (release 46, Nov 2019) using the Pathema Bioinformatics Resource Center (https://plasmodb.org/plasmo/) (Collaborative, 2001). Plasmodium Sar/Arf homologs were retrieved via the BLASTP algorithm using the human Arf1 and Sar1 GTPases as queries in the P. falciparum 3D7 database. (B) Sequence alignment of Homo sapiens Arf1 and Sar1 and six Plasmodium Sar1/Arf families of proteins. Amino acid sequences were aligned using Clustal Omega (EMBL-EBI, Welcome Genome Campus). The N-terminus myristoylation site with a glycine residue specific for the Arf family and the GTP-binding consensus boxes are indicated in blue and red boxes, respectively (Pereira-Leal and Seabra, 2000). The effector regions of the Arf/Arf-like (Arl) and Sar1 families are indicated in light blue and magenta, respectively. Note that PF3D7_1442000 and PF3D7_1316200 lack an N-terminal glycine residue. PF3D7_0920500 and PF3D7_1316200 are missing the second GTP-binding box. Two conserved residues, that are substituted to create constitutively active and inactive mutants, are indicated by black and white arrowheads, respectively. (C) Amino acid percentage identity matrix of Pf3D7_1020900 and the five human Arf1. [file Image_2.tiff]

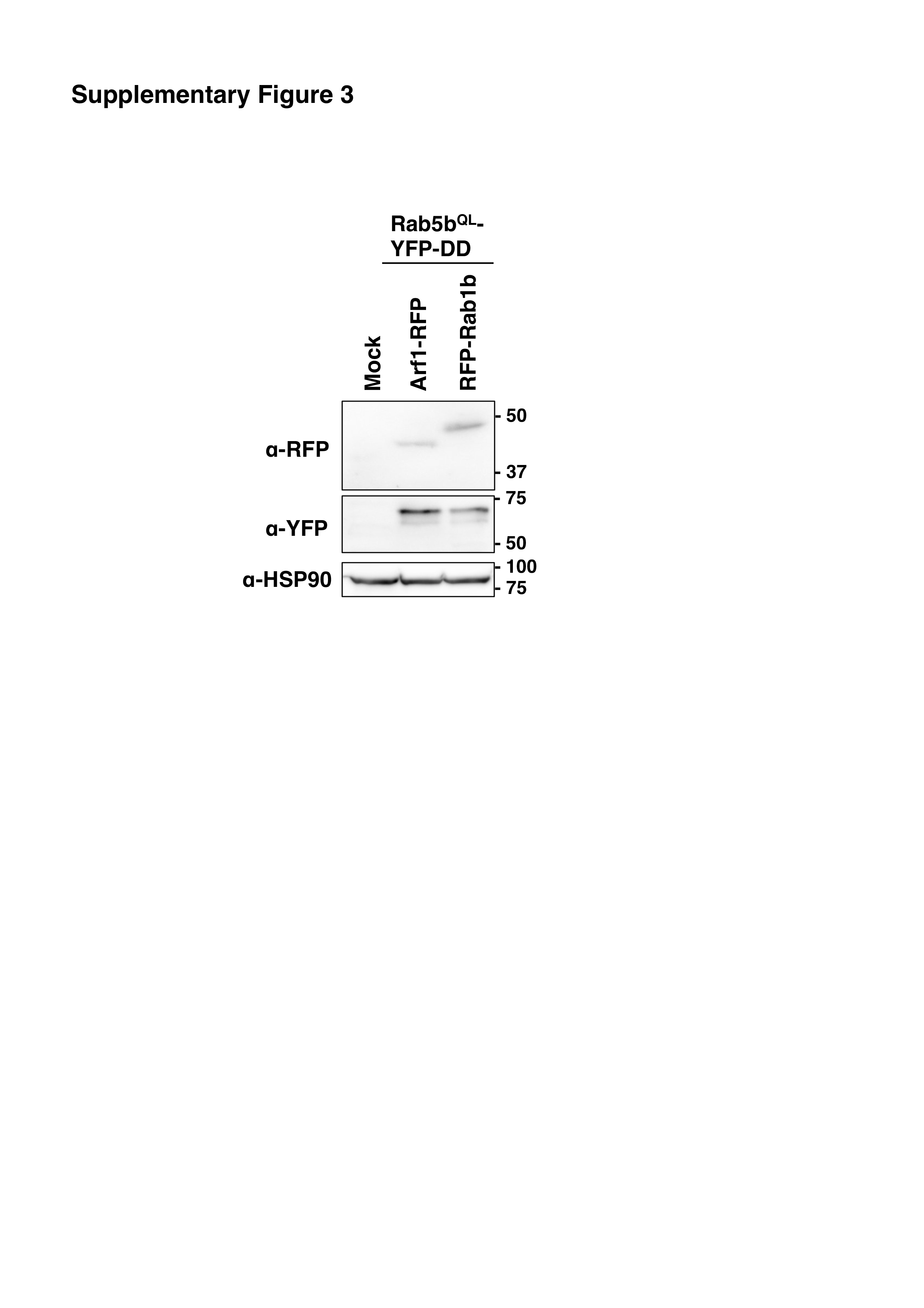

Supplement: Supplementary Figure 3 — Immunoblots showing the expression of full length of PfArf1-RFP, and RFP-PfRab1b. Parasite lysates expressing PfRab5bQ94L-YFP-DD and Arf1-RFP or RFP-PfRab1b were subjected to immunoblot analysis using anti-RFP and anti-GFP antibodies. Anti-Hsp90 antibody was used as a loading control. Mock is the non-transformant parasite. [file Image_3.tiff]

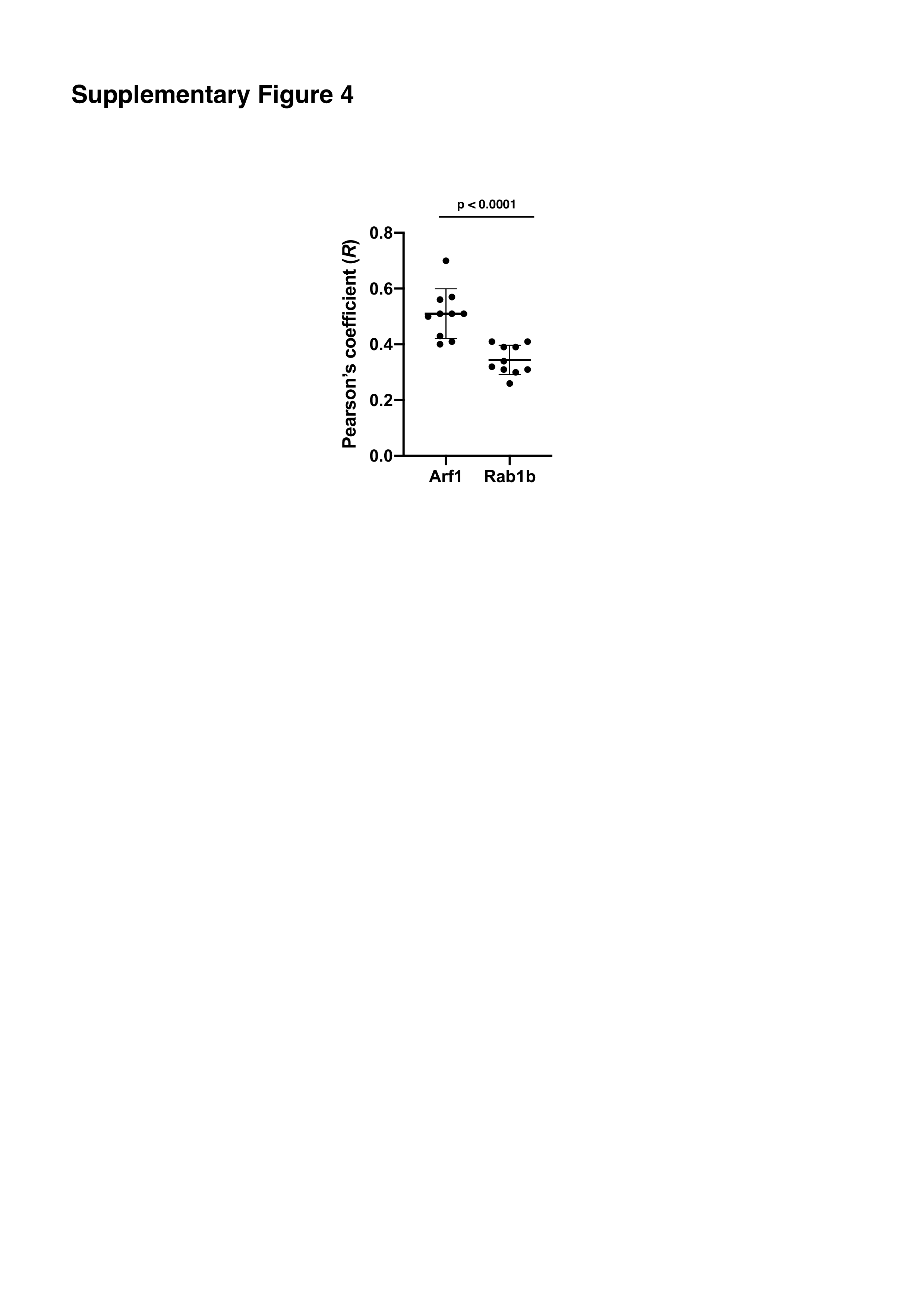

Supplement: Supplementary Figure 4 — Pearson’s correlation coefficient between PfRab5bQ94L-YFP-DD and PfArf1-RFP or RFP-PfRab1b. The fluorescence intensities of the merged YFP and RFP signals were analyzed using Fiji-Image J software to generate Pearson correlation coefficients. The scatter plot represents Pearson correlation coefficients values from ten independent parasites, which are obtained from three independent transfectants. The statistical significance was determined using Student’s t-test. [file Image_4.tiff]

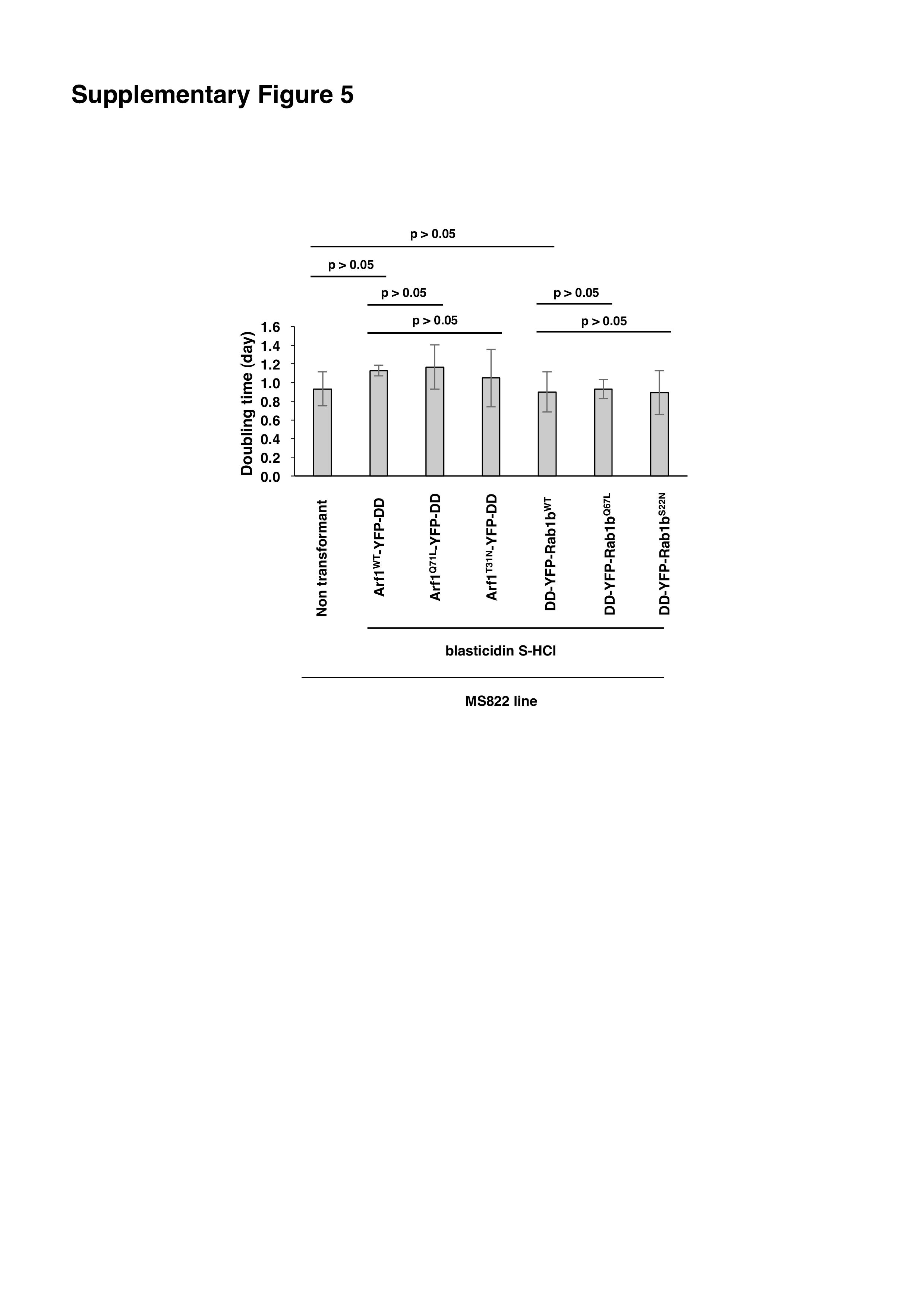

Supplement: Supplementary Figure 5 — Expressions of PfArf1 or PfRab1b mutants showed no effect on parasites growth. The population doubling time of non-transformant MS822 line, PfArf1 and PfRab1b mutants were measured for 2 days cultivation from three independent assays. The average (bar graph) and the standard deviation (thin bar) are indicated. Mock was cultured without drugs and PfArf1, PfRab1b mutants were cultured with BSD. Except from MS822 line, transformed parasite were cultured in the presence of 2.5 µg/ml of BSD and the expressions of PfArf1 and PfRab1b mutants were stabilized by 0.5 µM Shld1. A statistical significance was evaluated using Student’s t-test. [file Image_5.tiff]
